# Circ_0024390 promotes renal tubular EMT through the miR-6890-3p/CHD4 axis: implications for renal interstitial fibrosis

**DOI:** 10.3389/fphar.2026.1941987

**Published:** 2026-10-14

**Authors:** Xue Yang, Lujie Zhang, Yan Song, Gang Liu

**Affiliations:** 1 Department of Nephrology, Multidisciplinary Innovation Center for Nephrology, The Second Qilu Hospital of Shandong University, Jinan, Shandong, China; 2 Nephrology Research Institute of Shandong University, Jinan, Shandong, China; 3 Key Laboratory of Reproductive Endocrinology of Ministry of Education, Shandong University, Jinan, Shandong, China

**Keywords:** CHD4, circ_0024390, epithelial-mesenchymal transition, miR-6890-3p, renal interstitial fibrosis

## Abstract

**Background:**

Renal interstitial fibrosis (RIF) is a central pathological event in the progression of various chronic kidney diseases (CKD) to end-stage renal disease, for which effective therapeutic strategies remain lacking. Epithelial-mesenchymal transition (EMT) of renal tubular epithelial cells serves as a pivotal mechanism driving RIF. While circular RNAs (circRNAs) have been implicated in various pathological processes, their specific roles and underlying mechanisms in the progression of RIF remain incompletely elucidated.

**Methods:**

A circRNA microarray was utilized to screen differentially expressed circRNAs in a TGF-β1-induced HK-2 cell model of renal fibrosis. Loss-of-function experiments were performed to explore the biological effects of circ_0024390 on renal tubular EMT, as well as the proliferation and migration of HK-2 cells. Mechanistic assays were further conducted to verify the competing endogenous RNA (ceRNA) regulatory mechanism of circ_0024390.

**Results:**

Circ_0024390 was significantly upregulated in the in vitro fibrosis model, specifically in TGF-β1-induced HK-2 cells. Functional studies demonstrated that knockdown of circ_0024390 effectively attenuated renal tubular EMT and inhibited key cellular processes, including proliferation and migration. Mechanistic investigations further revealed that circ_0024390 functioned as a ceRNA by sponging miR-6890-3p. This sponging relieved the repressive effect of miR-6890-3p on its downstream target, chromodomain helicase DNA-binding protein 4 (CHD4), leading to the upregulation of CHD4 expression.

**Conclusion:**

These findings delineate a novel circ_0024390/miR-6890-3p/CHD4 axis that promotes EMT in renal tubular epithelial cells and contributes to the progression of RIF. This study provides new insights into the pathogenesis of RIF and highlights circ_0024390 as a potential promising therapeutic target for CKD.

## 1 Introduction

Chronic kidney disease (CKD) is a global public health concern, characterized by high prevalence and mortality rates, affecting approximately 10%–14% of the worldwide population (Humphreys, 2018; Glassock et al., 2017). Renal interstitial fibrosis (RIF) represents a common pathological process across various forms of CKD and plays a critical role in the progression to end-stage renal disease (ESRD) (Tsai et al., 2010; Huang et al., 2023). A hallmark of RIF is the excessive deposition of extracellular matrix (ECM) proteins, primarily collagen I and III, which leads to the expansion of the interstitial space between the renal tubular basement membrane and adjacent capillaries (Xu et al., 2022). Despite increasing research efforts, effective strategies for the early diagnosis and treatment of RIF remain limited (Grams et al., 2013).

Epithelial-mesenchymal transition (EMT) is a key pathogenic mechanism underlying RIF, characterized by the phenotypic transformation of renal tubular epithelial cells into myofibroblasts (Sheng and Zhuang, 2020). During this transformation, renal epithelial cells exhibit a decreased expression of adhesion proteins, such as E-cadherin, while concurrently upregulating the expression of fibroblast markers, including vimentin and α-smooth muscle actin (α-SMA) (Kriz et al., 2011). Extensive research has identified transforming growth factor-β1 (TGF-β1) as a central mediator of this process, capable of inducing the conversion of renal tubular epithelial cells to myofibroblasts through EMT, thereby promoting renal fibrosis (Zou et al., 2022). As a crucial pro-fibrotic cytokine, TGF-β1 plays a pivotal role in both EMT and the progression of RIF (Sime and O'Reilly, 2001; Li et al., 2019). Although TGF-β1 is a well-established mediator of EMT, the precise downstream molecular mechanisms contributing to fibrotic remodeling remain incompletely understood. Thus, elucidating the mechanisms underlying EMT and RIF, and identifying key molecular targets regulating EMT in renal tubular epithelial cells, is critical for developing effective strategies to prevent and treat RIF.

Circular RNAs (circRNAs) are a class of endogenous non-coding RNAs primarily generated from pre-mRNAs through alternative splicing events (Memczak et al., 2013). Characterized by their stable circular structure and highly conserved sequences across species, circRNAs play significant roles in transcriptional and post-transcriptional regulation, and participate in diverse biological processes (Cheng et al., 2022). For example, circRNAs can act as competing endogenous RNAs (ceRNAs), binding to intracellular microRNAs (miRNAs) and thereby modulating gene expression by preventing miRNAs from targeting their downstream messenger RNAs (mRNAs) (Zhang et al., 2019). In addition to these functions, circRNAs are involved in regulating other RNAs and protein activity (Zhang and Xin, 2018). Furthermore, accumulating evidence indicates that circRNA expression patterns are dysregulated in a range of diseases, including ischemic heart disease (Zeng et al., 2017), cancer (Ju et al., 2019), diabetes (Fang et al., 2018), and lupus nephritis (Ouyang et al., 2018). Consequently, circRNAs hold great potential for the development of new diagnostic and therapeutic methods for various diseases, highlighting their significant research value. Recent studies have found that some circRNAs are closely related to fibrotic diseases, as they can influence key pathological processes (Shi et al., 2024; Zhou et al., 2024). For example, certain circRNAs are involved in fibrosis of the lungs, liver, and heart, mainly by affecting the EMT process (Li et al., 2018; Chen et al., 2017; Tang et al., 2017). Despite these findings, the precise role of circRNAs in regulating EMT, particularly in RIF, remains poorly understood.

In this study, we hypothesized that circRNAs may be involved in the development of RIF by interacting with miRNAs. To better understand the pathogenesis of RIF, we investigated circRNA and miRNA expression profiles and explored their regulatory relationship with chromodomain helicase DNA-binding protein 4 (CHD4).

## 2 Materials and methods

### 2.1 Cell culture and treatments

The HK-2 cell line was purchased from the American Type Culture Collection (ATCC) and cultured in DMEM/F12 (BasalMedia, China) supplemented with 10% fetal bovine serum (FBS; Lonsera, Uruguay) and 1% antibiotics (penicillin and streptomycin, BasalMedia, China) at 37 °C with 5% CO_2_ in a humidified incubator. To induce EMT, HK-2 cells were first cultured in serum-free medium for 6 h and then treated with TGF-β1 (10 ng/mL, R&D Systems, United States) for 12 h. Additionally, human embryonic kidney 293T cells (HEK293T), a kind gift from Dr. Chengyun Zheng (Shandong University), were cultured in DMEM (BasalMedia, China) with 10% FBS and 1% penicillin-streptomycin at 37 °C in a 5% CO_2_ incubator.

### 2.2 CircRNA microarray analysis

Total RNA was extracted from TGF-β1-induced and control HK-2 cells using TRIzol reagent (Invitrogen, United States), and circRNA expression profiling was performed using the Clariom D Human microarray (Affymetrix, United States) according to the manufacturer’s instructions. Differentially expressed circRNAs were identified using a cutoff of |fold change| > 1.2 and p-value <0.05. Among them, circ_0024390 was selected for further validation and mechanistic analysis due to its significant upregulation and potential functional relevance to EMT.

### 2.3 Cell transfection

Cells were cultured in medium without antibiotics before transfection. Small interfering RNA (siRNA) targeting circ_0024390 (designed and synthesized by Tsingke Biol, China) or negative control siRNA was transfected into HK-2 cells using the Lipofectamine 2000 reagent (Invitrogen, United States) according to the manufacturer’s instructions. After 6 h of transfection, the culture medium was replaced with fresh medium, with or without TGF-β1 for an additional 12 h incubation. The silencing efficiency of circ_0024390 was confirmed by RT-qPCR after transfection. Similarly, the siRNA targeting CHD4 (designed and synthesized by General Biol, China) was also transfected using the same method, and knockdown efficiency was validated by RT-qPCR. The sequences of siRNAs are shown in Table 1. In addition, HK-2 cells were transfected with miR-6890-3p mimic or inhibitor, as well as the corresponding negative controls (all designed and synthesized by General Biol, China) using the same reagent. Corresponding negative controls were included in all experiments to account for non-specific effects of transfection.

**TABLE 1:** siRNA sequences.

siRNA	Sequence
siRNA-circ_0024390-1-sense	CUG​GUU​UAU​GAA​AUU​CUG​A/TT
siRNA-circ_0024390-1-antisense	UCA​GAA​UUU​CAU​AAA​CCA​G/TT
siRNA-circ_0024390-2-sense	GGU​UUA​UGA​AAU​UCU​GAG​C/TT
siRNA-circ_0024390-2-antisense	GCU​CAG​AAU​UUC​AUA​AAC​C/TT
siRNA-circ_0024390-3-sense	UUU​AUG​AAA​UUC​UGA​GCA​A/TT
siRNA-circ_0024390-3-antisense	UUG​CUC​AGA​AUU​UCA​UAA​A/TT
siRNA-CHD4-1-sense	GAA​AGA​AGA​AGA​AGA​GAA​A/TT
siRNA-CHD4-1-antisense	UUU​CUC​UUC​UUC​UUC​UUU​C/TT
siRNA-CHD4-2-sense	AGA​AAG​AGU​UCA​AGG​CAU​A/TT
siRNA-CHD4-2-antisense	UAU​GCC​UUG​AAC​UCU​UUC​U/TT
siRNA-CHD4-3-sense	GGA​CCU​GAA​UGA​UGA​GAA​A/TT
siRNA-CHD4-3-antisense	UUU​CUC​AUC​AUU​CAG​GUC​C/TT
Non-targeting siRNA sense	UUC​UCC​GAA​CGU​GUC​ACG​U/TT
Non-targeting siRNA antisense	ACG​UGA​CAC​GUU​CGG​AGA​A/TT

### 2.4 Real-time quantitative PCR (RT-qPCR)

Total RNA was isolated from HK-2 cells using TRIzol reagent (Invitrogen, United States). Complementary DNA (cDNA) was then synthesized following the protocol of the Strand cDNA Synthesis SuperMix for qPCR kit (Yeasen Biotechnology, China). RT-qPCR was performed on a QuantStudio 5 Real-Time PCR System (Thermo Fisher Scientific, United States) using a SYBR Green Master Mix kit (Yeasen Biotechnology, China). β-Actin was used as the internal control for quantifying circRNA and mRNA expression, while U6 was employed as the internal control for miRNA expression. Relative expression levels of RNAs were quantified using the 2^−ΔΔCT^ method. All primer sequences are listed in Table 2.

**TABLE 2:** Primer sequences for RT-qPCR.

Primers	Sequence (5′-3′)
circ_0060973-F	AGG​CCT​GAA​GAA​ACT​GCT​TGT
circ_0060973-R	CTG​CAG​GTA​CGG​ATA​GGT​GG
circ_0034411-F	CGT​CAC​CTC​AGA​CTG​CCT​AC
circ_0034411-R	GTC​GGT​GCA​CTC​TCC​ATA​GG
circ_0034417-F	GAC​TAG​GCG​TCC​TGT​TCC​TG
circ_0034417-R	GTC​CGG​AGT​AGA​GGT​TGC​TC
circ_0024390-F	AGA​ATG​ATG​GGC​ACT​ACC​GTG
circ_0024390-R	ACC​ATG​GAG​GGT​GGG​TTC​TC
TAGLN-F	TCC​AGA​CTG​TTG​ACC​TCT​TTG​AA
TAGLN-R	GCT​CCT​GCG​CTT​TCT​TCA​TAA​AC
miR-6890-3p-F	ACT​GCC​TAT​GCC​CCA​CAG​AA
miR-6890-3p-R	Universal reverse primer
U6-F	CTCGCTTCGGCAGCACA
U6-R	AAC​GCT​TCA​CGA​ATT​TGC​GT
CHD4-F	CAT​CCA​GAA​TGA​CCC​ACG​CT
CHD4-R	ACT​TTC​CGC​CAA​ACA​CTC​CA
β-actin-F	CCT​TCC​TGG​GCA​TGG​AGT​C
β-actin-R	TGA​TCT​TCA​TTG​TGC​TGG​GTG

### 2.5 Western blot analysis

Total protein was extracted from HK-2 cells using RIPA lysis buffer. Protein samples were then separated by SDS-PAGE and transferred onto PVDF membranes. The membranes were incubated overnight at 4 °C with primary antibodies targeting E-cadherin (1:1,000; Abcam, United States), α-SMA (1:1000; Abcam, United States), Fibronectin (1:1,000; Abcam, United States), Collagen III (1:1,000; Abcam, United States), N-cadherin (1:1,000; Abcam, United States), CHD4 (1:5,000; Abclonal, China), and β-tubulin (1:3,000; Cohesion, China). After washing, the membranes were incubated with HRP-conjugated secondary antibody (1:10,000; goat anti-rabbit or anti-mouse, Zsbio, China) for 1 h at room temperature. β-Tubulin was used as the internal control. Protein bands were visualized and analyzed using ImageJ software.

### 2.6 Cell viability assay

HK-2 cells were seeded in 96-well plates and incubated for 24 h. After that, the cells were treated with TGF-β1 for 12 h. Following treatment, 100 μL of fresh culture medium containing 10 μL of CCK-8 solution (Elabscience, China) was added to each well, and the cells were incubated at 37 °C. Cell viability was evaluated by measuring the absorbance at 450 nm using a microplate reader.

### 2.7 Transwell assay

A Transwell invasion assay was performed using a 24-well Transwell chamber. Briefly, 24 h after transfection, HK-2 cells (1 × 10^5^ cells/mL) were resuspended in serum-free medium and seeded into the upper chamber. Complete medium, with or without TGF-β1, was added to the lower chamber. The transwell plates were incubated at 37 °C in a 5% CO_2_ incubator for 12 h. Subsequently, non-migrated cells on the upper surface of the membrane were gently removed using a cotton swab. The migrated cells on the lower surface of the membrane were fixed with 4% paraformaldehyde for 20 min and stained with crystal violet (Beyotime, China) for an additional 15 min. Migrating cells were observed under an optical microscope (Olympus, Japan), and the migrated cells in three randomly selected fields of view per well were counted using ImageJ software.

### 2.8 Wound healing assay

Transfected HK-2 cells were prepared as a single-cell suspension, and 6 × 10^5^ cells/mL were seeded into each well of a 6-well plate. Upon reaching 80%–90% confluence, a cell-free gap was created by gently scraping the monolayer with a sterile 1-mL pipette tip (defined as 0 h). Images of the wound area were captured at 0 and 24 h using an inverted microscope (Olympus, Japan), and the wound area was quantified using ImageJ software. The wound healing rate was calculated as (A_0_ − A_24_)/A_0_ × 100%, where A_0_ and A_24_ denote the wound areas at 0 and 24 h, respectively.

### 2.9 EdU cell proliferation assay

Cell proliferation was detected using an EdU cell proliferation kit with Alexa Fluor 555 (Beyotime, China), following the manufacturer’s protocol. The cells were incubated with 10 μM EdU for 2 h. Subsequently, EdU was labeled with Alexa Fluor 555 via a click reaction, and then Hoechst 33,342 was used for cell nucleus staining. Images were acquired using an inverted fluorescence microscope (Olympus, Japan).

### 2.10 Bioinformatic prediction of the circ_0024390/miR-6890-3p/CHD4 regulatory axis

Potential miRNA targets of circ_0024390 were predicted using circBank (http://www.circbank.cn/) and miRDB (http://www.mirdb.org/). The intersection of the results from the two databases identified miR-6890-3p as the leading candidate, which was predicted to contain a complementary binding site for circ_0024390. To identify potential downstream targets of miR-6890-3p, miRDB and miRTarBase (https://mirtarbase.cuhk.edu.cn/) were queried. CHD4 was ultimately selected as the target gene, as it was consistently predicted in the GEO datasets GSE76882 and GSE261665 and has previously been reported to be involved in fibrosis (Shi et al., 2025; Luo et al., 2024; Sun et al., 2023).

### 2.11 Public datasets and pathway analysis

The datasets GSE76882 and GSE261665 were downloaded from the Gene Expression Omnibus (GEO) database (https://www.ncbi.nlm.nih.gov/geo/). R software (version 4.5.1) was used to convert row counts into transcripts per million (TPM) values, which were subsequently log2-transformed for downstream analyses. Gene set enrichment analysis (GSEA) was performed by grouping samples based on CHD4 expression levels, followed by calculation of log fold change (logFC) values for gene ranking. The enrichment scores were determined using the c5. Go. V2025.1. Hs. Symbols gene set for human samples and m5. Go. V2025.1. Mm. Symbols gene set for mouse samples from the Molecular Signature Database (MSigDB).

### 2.12 RNA FISH assay

RNA fluorescence *in situ* hybridization (FISH) was performed strictly according to the manufacturer’s instructions using an RNA FISH kit (GenePharma, China). Cells were fixed with 4% paraformaldehyde and hybridized overnight at 37 °C with RNA probes (GenePharma, China) in hybridization buffer. The circ_0024390 probe was labeled with FITC, and the miR-6890-3p probe was labeled with CY3. Cell nuclei were stained with DAPI. Images were acquired using a confocal microscope (Zeiss, Germany).

### 2.13 Dual-luciferase reporter assay

Dual-luciferase reporter assay was used to detect the interaction between miR-6890-3p and circ_0024390.293T cells seeded in 24-well plates were transfected with miR-6890-3p mimic or a negative control (NC; General Biol, China). Additionally, cells were co-transfected with plasmids containing either wild-type or mutant circ_0024390 (General Biol, China). After 48 h, luciferase activity was measured using a Dual Luciferase Reporter Gene Assay Kit (Yeasen Biotechnology, China) according to the manufacturer’s instructions.

Similarly, to detect the interaction between miR-6890-3p and CHD4, a dual-luciferase reporter assay was performed. 293T cells were co-transfected with miR-6890-3p mimic, mimic NC, wild-type CHD4 plasmid, or mutant CHD4 plasmid (General Biol, China). After 48 h, luciferase activity was measured using the same Dual Luciferase Reporter Gene Assay Kit (Yeasen Biotechnology, China).

### 2.14 Statistical analysis

All data are presented as the mean ± standard error of the mean (SEM). Comparisons between two groups were performed using a Student’s t-test. One-way analysis of variance (ANOVA) with Bonferroni’s post-test was used to analyze differences among multiple groups. All statistical analyses were conducted using GraphPad Prism 8.0 software. Statistical significance was defined as follows: *p < 0.05, **p < 0.01, and ***p < 0.001.

## 3 Results

### 3.1 Circ_0024390 expression is elevated in TGF-β1-stimulated HK-2 cells

Upon stimulation with 10 ng/mL TGF-β1 (Xiao et al., 2024; Chen et al., 2023; Liu et al., 2021; Luo et al., 2023), HK-2 cells underwent a morphological change from a cobblestone-like shape to an elongated spindle shape (Figure 1A). The circRNA expression profiles of TGF-β1-stimulated HK-2 cells and the control group were evaluated using microarray hybridization. The variation in circRNA expression between the two groups is shown in Supplementary Table S1. A total of 1,658 differentially expressed circRNAs with a fold change greater than 1.2 were identified. Hierarchical clustering analysis was conducted based on these 1,658 differentially expressed circRNAs (Figure 1B). The four circRNAs exhibiting the most significant differential expression were selected for validation by RT-qPCR. The results confirmed that the expression of circ_0024390 was markedly increased in TGF-β1-stimulated HK-2 cells compared to the control group, consistent with microarray results (Figure 1C). However, the expression of circ_0034417, circ_0060973, and circ_0034411 was inconsistent with microarray results, possibly due to technical variability (Figure 1C). Further analysis revealed that TAGLN was susceptible to RNase R digestion in HK-2 cells, whereas circ_0024390 remained resistant to RNase R treatment (Figure 1D). In addition, circ_0024390 was amplified using divergent primers in cDNA but not in genomic DNA (gDNA) (Figure 1E). These data collectively indicate that circ_0024390 is circular.

**FIGURE 1:**
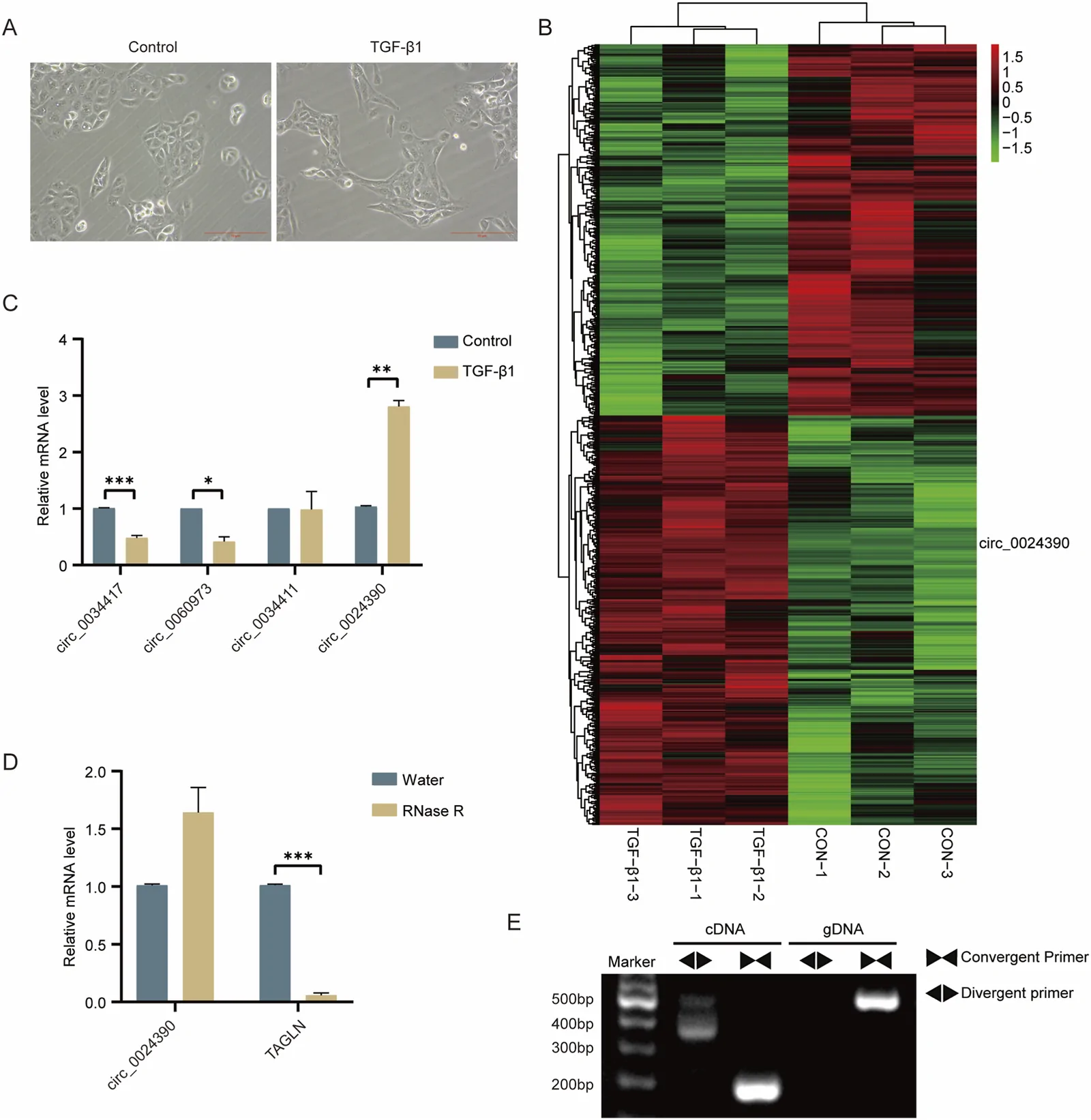
CircRNA expression profile in TGF-β1-stimulated HK-2 cells. **(A)** Morphology changes of HK-2 cells following TGF-β1 stimulation (n = 3, scale bar, 10 μm). **(B)** Hierarchical clustering heatmap illustrating differentially expressed circRNAs with an expression fold change greater than 1.2. Red and green colors represent high and low expression levels, respectively (n = 3). **(C)** Validation of four differentially expressed circRNAs using RT-qPCR (n = 3) **(D)**. RT-qPCR analysis of circ_0024390 and TAGLN levels in HK-2 cells following RNase R treatment (n = 3). **(E)** Agarose gel electrophoresis experiment showed that divergent primers specifically amplified circ_0024390 from cDNA but not from genomic DNA (gDNA) in HK-2 cells (n = 3). Results are representative of three independent experiments. Data are presented as mean ± SEM. *p < 0.05, **p < 0.01, ***p < 0.001, Student’s t test for **(C,D)**.

### 3.2 Circ_0024390 promotes the EMT of HK-2 cells

HK-2 cells were transfected with siRNA targeting circ_0024390 and treated with or without 10 ng/mL TGF-β1 for 12 h. RT-qPCR confirmed effective silencing of circ_0024390 expression by siRNA (Figure 2A). Cell viability was assessed using CCK-8 assay following TGF-β1 stimulation at different time points. To ensure that observed EMT effects were not due to changes in cell viability, we confirmed that a 12 h stimulation with 10 ng/mL TGF-β1 did not significantly affect cell viability. However, longer incubation periods of 24, 48, and 72 h significantly reduced cell viability (Figure 2B). To evaluate cell proliferation, an EdU assay was performed to monitor DNA replication activity. TGF-β1 treatment significantly enhanced DNA replication, whereas the number of EdU-positive cells in the siRNA circ_0024390 group was significantly lower compared to the TGF-β1-treated group (Figure 2C). Transwell and wound-healing assays were conducted to investigate the impact of circ_0024390 on HK-2 cell migration. The results demonstrated that circ_0024390 knockdown significantly inhibited the migratory ability of HK-2 cells (Figures 2D,E). Western blot analysis further revealed that circ_0024390 knockdown decreased the expression of Fibronectin, Collagen III, N-cadherin, and α-SMA, while increasing the expression of E-cadherin (Figure 2F). Collectively, these results confirm that circ_0024390 promotes EMT in HK-2 cells.

**FIGURE 2:**
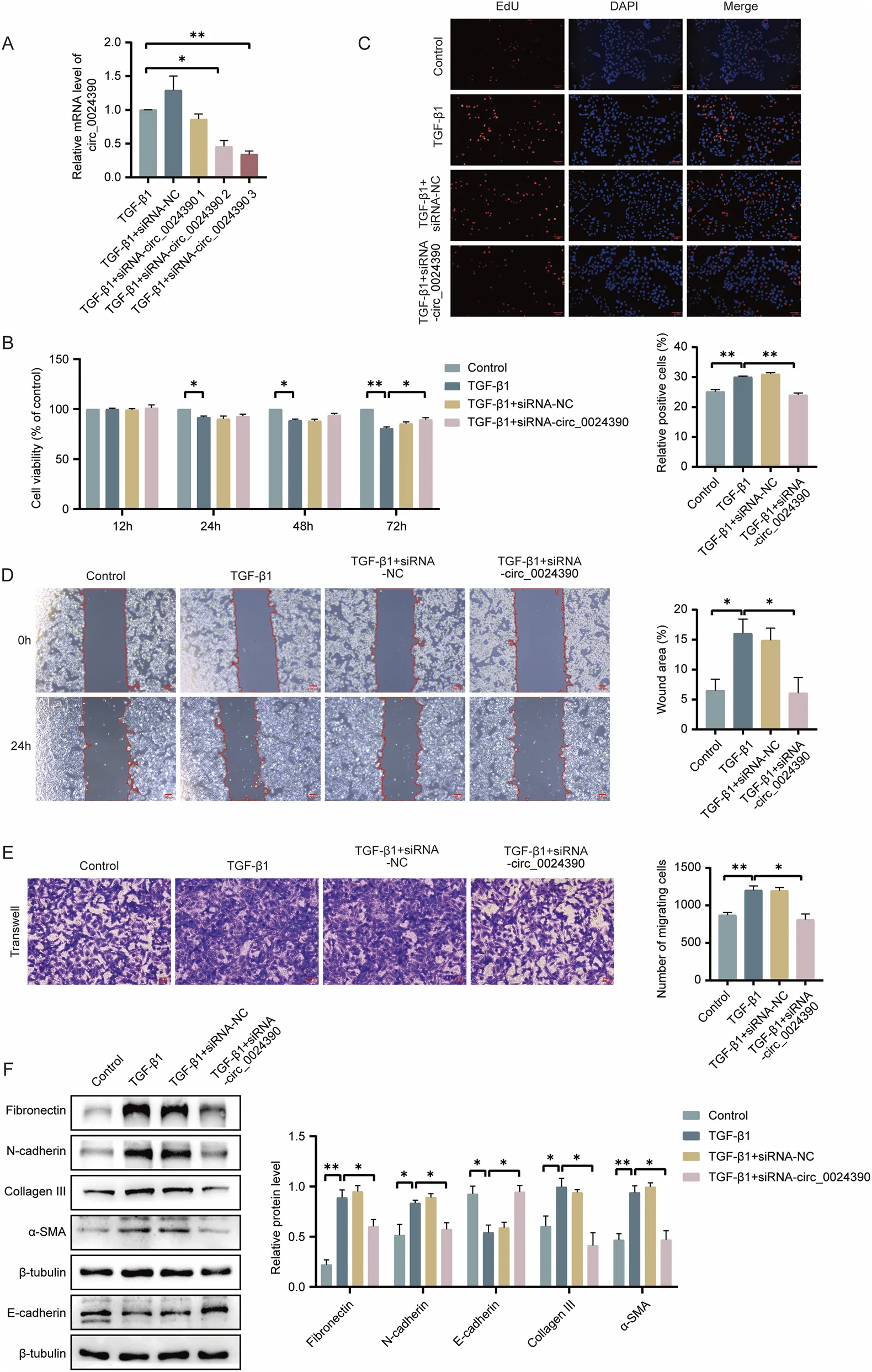
Circ_0024390 regulates proliferation, migration, and EMT of HK-2 cells. **(A)** RT-qPCR analysis of circ_0024390 expression in HK-2 cells transfected with siRNA targeting circ_0024390 (n = 3). **(B)** CCK-8 assay evaluating the effect of siRNA targeting circ_0024390 on the viability of HK-2 cells following TGF-β1 stimulation (n = 3). **(C)** EdU assay assessing the proliferation of HK-2 cells under different treatments (n = 3; scale bar, 100 μm). **(D)** Wound healing assay evaluating the effect of siRNA targeting circ_0024390 on the migration ability of HK-2 cells (n = 3; scale bar, 100 μm). **(E)** Transwell assay evaluating the migration ability of HK-2 cells under different treatments (n = 3; scale bar, 100 μm). **(F)** Representative Western blot images and quantitative analysis of Fibronectin, N-cadherin, E-cadherin, Collagen III, and α-SMA protein levels (n = 3). Data are presented as mean ± SEM. *p < 0.05, **p < 0.01, ***p < 0.001, one-way ANOVA with Bonferroni’s post-test **(A–F)**.

### 3.3 Circ_0024390 functions as a ceRNA to sponge miR-6890-3p

CircRNAs frequently function as ceRNAs, binding to specific miRNAs and thereby releasing their suppression on downstream mRNA targets. This ceRNA mechanism is a common way by which circRNAs regulate cellular processes, including fibrosis-related pathways. Considering this possibility, we used bioinformatics tools (circBank and miRDB) to predict the potential target miRNAs of circ_0024390. Intersection analysis identified miR-6890-3p as the most likely complementary miRNA to circ_0024390 (Figure 3A). A dual-luciferase reporter assay was conducted to validate the interaction between circ_0024390 and miR-6890-3p. The results showed that miR-6890-3p significantly reduced the luciferase activity in the presence of wild-type circ_0024390, but had no effect on that of the mutant circ_0024390 (Figure 3B). Additionally, FISH analysis revealed that circ_0024390 mainly interacts with miR-6890-3p in the cytoplasm of HK-2 cells (Figure 3C). Moreover, the expression level of miR-6890-3p was increased in HK-2 cells transfected with siRNA targeting circ_0024390 (Figure 3D). In conclusion, circ_0024390 functions as a ceRNA by sponging miR-6890-3p, thereby regulating its expression level in HK-2 cells.

**FIGURE 3:**
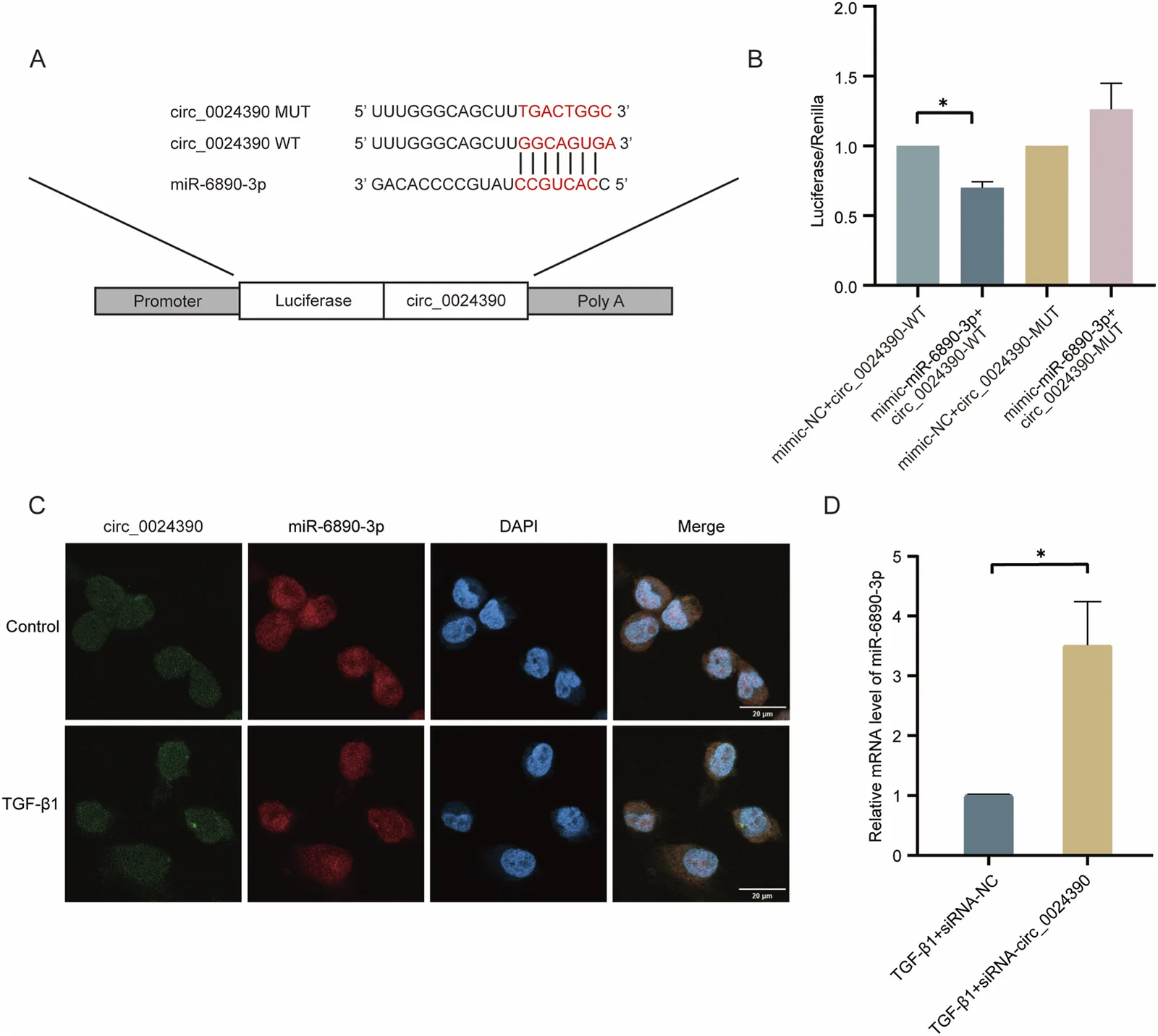
MiR-6890-3p is a direct target of circ_0024390. **(A)** circ_0024390 contains a complementary binding sequence for miR-6890-3p. **(B)** Dual-luciferase reporter assays were conducted in 293T cells transfected with circ_0024390-WT or circ_0024390-MUT vectors, along with miR-6890-3p mimic or negative control (NC) (n = 3). **(C)** RNA fluorescence *in situ* hybridization (FISH) was performed to detect the co-localization of circ_0024390 (green) and miR-6890-3p (red) in HK-2 cells treated with TGF-β1 (n = 3; scale bar, 20 μm). **(D)** RT-qPCR was used to measure miR-6890-3p expression levels in HK-2 cells transfected with siRNA targeting circ_0024390 (n = 3). Data are presented as mean ± SEM. *p < 0.05, **p < 0.01, ***p < 0.001, one-way ANOVA with Bonferroni’s post-test **(B)**, or Student’s t test **(D)**.

### 3.4 MiR-6890-3p mimic inhibits EMT in HK-2 cells

The role of miR-6890-3p in regulating EMT in HK-2 cells was investigated. RT-qPCR showed that miR-6890-3p mimic significantly increased the expression of miR-6890-3p, while miR-6890-3p inhibitor decreased its expression (Figure 4A). The CCK-8 assay confirmed that TGF-β1 treatment significantly reduced the cell viability of HK-2 cells at 24 h, 48 h, and 72 h, while miR-6890-3p mimic had no significant effect on cell viability (Figure 4B). Next, the effect of miR-6890-3p mimic on cell proliferation and migration were evaluated. Transwell and wound-healing assays showed that miR-6890-3p mimic significantly inhibited cell migration (Figures 4C,D). Similarly, EdU assays demonstrated that the number of EdU-positive cells in the miR-6890-3p mimic group was significantly lower than that in the TGF-β1 group (Figure 4E). Western blot analysis showed that miR-6890-3p mimic reduced the expression of Fibronectin, Collagen III, N-cadherin, and α-SMA, while increasing the expression of E-cadherin (Figure 4F). To further confirm whether the pro-fibrotic effect of circ_0024390 is mediated by miR-6890-3p, rescue experiments were conducted. Transfection with miR-6890-3p inhibitor increased the expression of Fibronectin, Collagen III, N-cadherin, and α-SMA, reversing the suppression induced by circ_0024390 knockdown. Additionally, miR-6890-3p inhibitor decreased the expression of E-cadherin, reversing the increase caused by circ_0024390 knockdown (Figure 4G). These results demonstrate that circ_0024390 promotes EMT in HK-2 cells in a manner dependent on miR-6890-3p.

**FIGURE 4:**
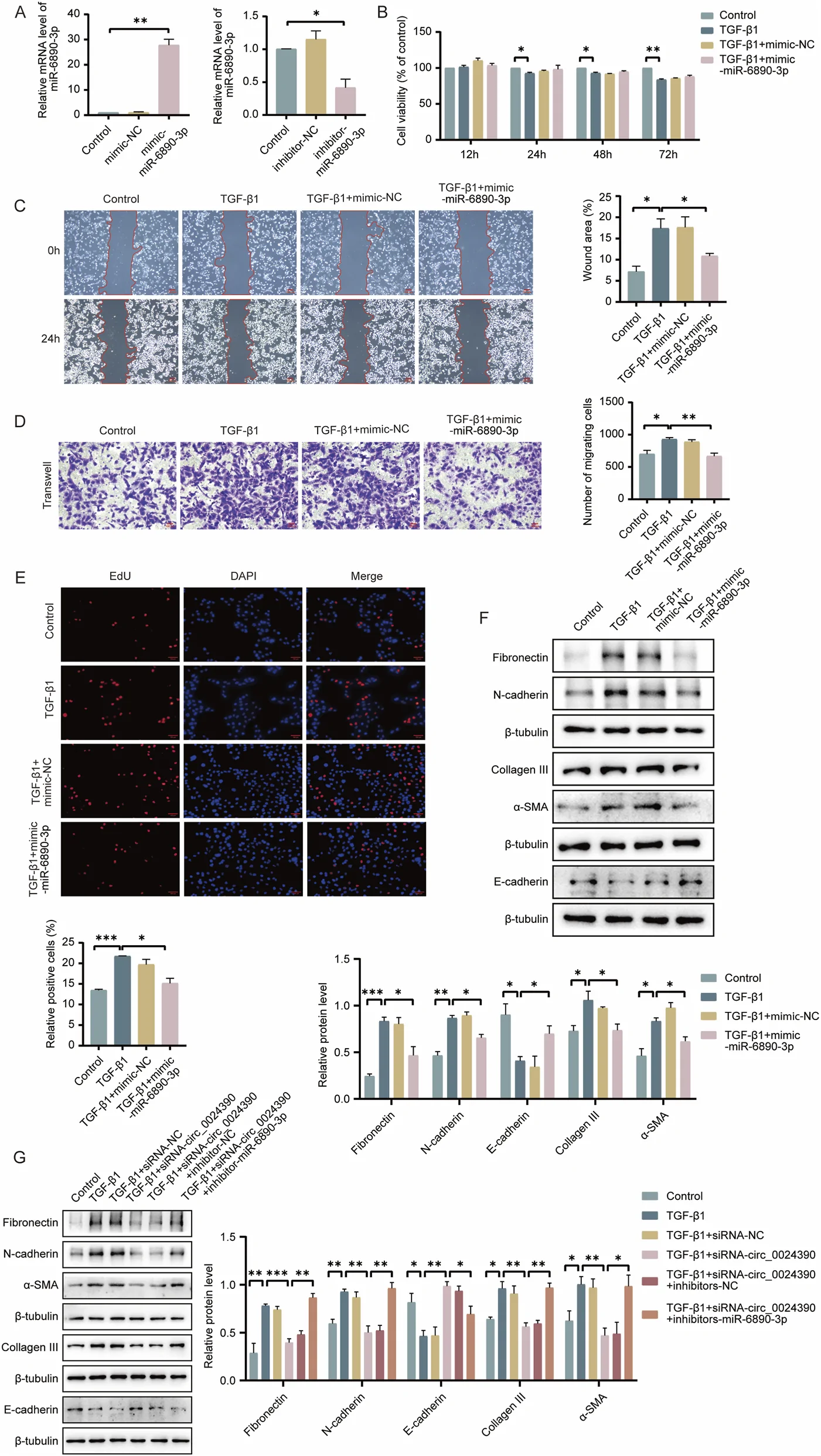
Circ_0024390 promotes EMT by sponging miR-6890-3p. **(A)** RT-qPCR was performed to detect the level of miR-6890-3p in HK-2 cells transfected with miR-6890-3p mimic or inhibitor (n = 3). **(B)** CCK-8 assay was used to evaluate the effect of miR-6890-3p mimic on the viability of HK-2 cells treated with TGF-β1 (n = 3). **(C)** Wound healing assay was conducted to evaluate the migration ability of HK-2 cells transfected with miR-6890-3p mimic (n = 3; scale bar, 100 μm). **(D)** Transwell assay was performed to investigate the effect of miR-6890-3p mimic on the migration capacity of HK-2 cells (n = 3; scale bar, 100 μm). **(E)** EdU assay was utilized to assess the proliferation of HK-2 cells under different treatments (n = 3; scale bar, 100 μm). **(F)** Representative Western blot images and quantitative analysis of Fibronectin, N-cadherin, E-cadherin, Collagen III, and α-SMA protein levels (n = 3). **(G)** Rescue experiments showed that the circ_0024390 promotes EMT in a manner dependent on miR-6890-3p (n = 3). Data are presented as mean ± SEM. *p < 0.05, **p < 0.01, ***p < 0.001, one-way ANOVA with Bonferroni’s post-test **(A–G)**.

### 3.5 Downregulation of miR-6890-3p by circ_0024390 promotes EMT through upregulating CHD4

The potential target mRNAs of miR-6890-3p were identified using the miRDB and miRTarBase databases (Figure 5A). Intersection analysis and literature review revealed a potential binding site for miR-6890-3p in the 3′UTR region of CHD4 (Figure 5B). Dual-luciferase reporter assay showed that miR-6890-3p significantly inhibited luciferase activity of the wild-type CHD4 3′UTR, while no effect was observed on the luciferase activity of the mutant CHD4 3′UTR (Figure 5C). MiR-6890-3p mimic significantly reduced CHD4 mRNA and protein expression levels (Figures 5D,E). Furthermore, bioinformatics analysis of publicly available datasets from kidneys of interstitial fibrosis and tubular atrophy (IFTA) patients (GSE76882) and from unilaterally ureteral obstruction (UUO) mouse models (GSE261665), revealed that CHD4 expression levels were markedly upregulated in fibrotic kidneys tissues compared to controls (Figures 5F,H). GSEA results revealed that CHD4 was significantly enriched in gene sets associated with the EMT and ECM (Figures 5G,I). Given that ECM remodeling plays a crucial role in facilitating the EMT process (Scott et al., 2019; Yang et al., 2020), these findings suggest that CHD4 may contribute to renal fibrosis through EMT-mediated mechanisms. To further investigate the role of CHD4 in EMT, siRNA targeting CHD4 was transfected into HK-2 cells. RT-qPCR analysis confirmed effective knockdown of CHD4 expression (Figure 6A). Western blot analysis revealed that CHD4 knockdown reduced the expression of Fibronectin, Collagen III, N-cadherin, and α-SMA, while increasing the expression of E-cadherin (Figure 6B), indicating that CHD4 promotes EMT in HK-2 cells and exacerbates renal fibrosis. Based on these findings, we conclude that CHD4 is a direct target of miR-6890-3p. Additionally, circ_0024390 knockdown reduced CHD4 mRNA and protein expression levels (Figures 6C,D). Transfection with miR-6890-3p inhibitor increased CHD4 expression, thus reversing the reduction caused by circ_0024390 knockdown (Figure 6D). Collectively, these results indicate that circ_0024390 promotes EMT in HK-2 cells by downregulating miR-6890-3p, leading to upregulated CHD4 expression, which contributes to the progression of RIF.

**FIGURE 5:**
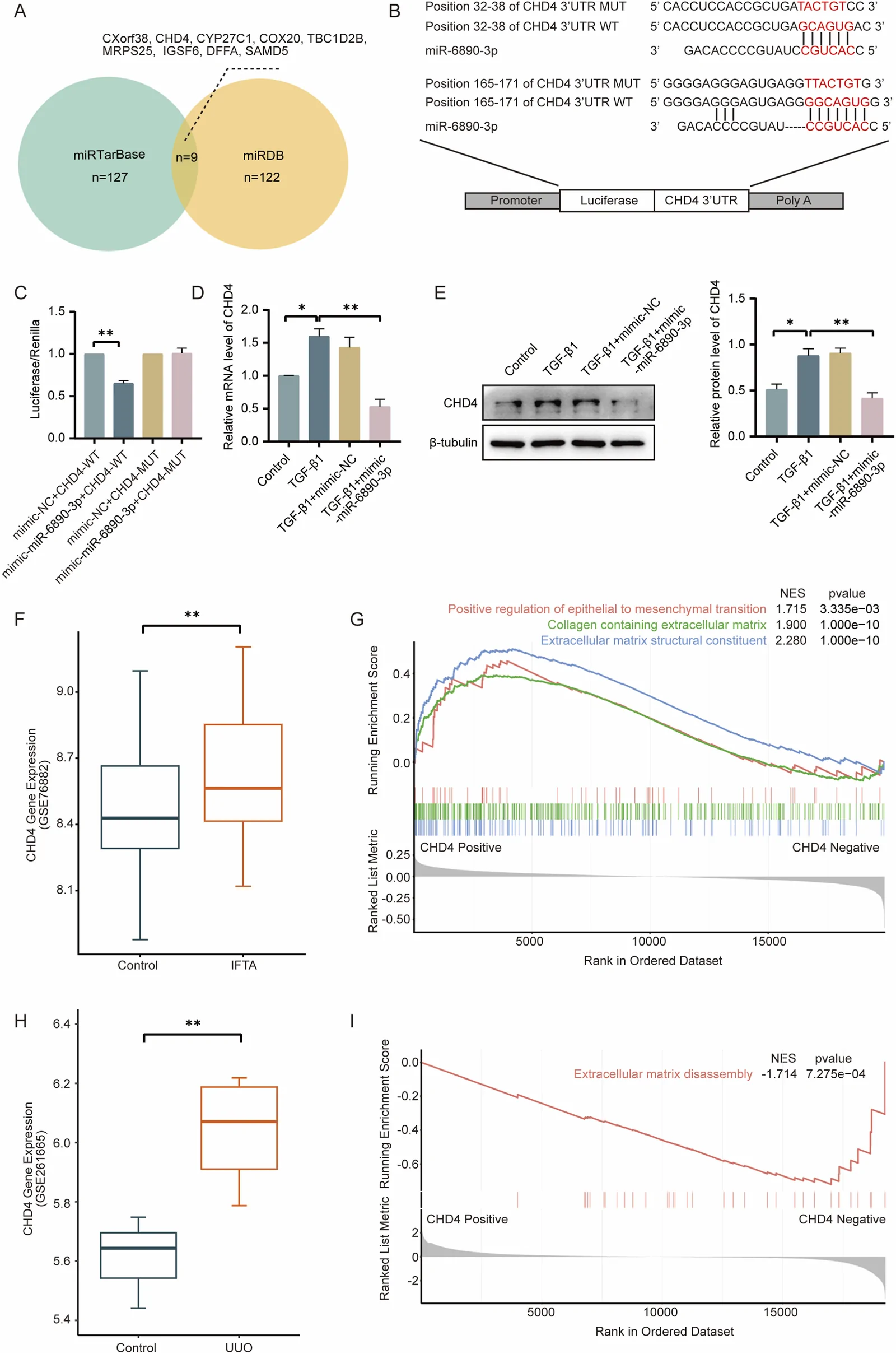
CHD4 is a direct target of miR-6890-3p. **(A)** Venn diagram showing the predicted target genes of miR-6890-3p identified using two online databases. **(B)** The 3′UTR of CHD4 mRNA contains a complementary binding sequence for miR-6890-3p. **(C)** Dual-luciferase reporter assays were performed in 293T cells transfected with CHD4-WT or CHD4-MUT vectors, along with miR-6890-3p mimic or negative control (NC) (n = 3). **(D)** RT-qPCR was performed to measure CHD4 mRNA levels in HK-2 cells transfected with miR-6890-3p mimic (n = 3). **(E)** Representative Western blot images and quantitative analysis of CHD4 protein levels in HK-2 cells transfected with miR-6890-3p mimic (n = 3). **(F)** CHD4 gene expression levels in dataset from kidneys of interstitial fibrosis and tubular atrophy (IFTA) patients (GSE76882). **(G)** Gene set enrichment analysis (GSEA) enrichment plot of CHD4 target genes (GSE76882). **(H)** CHD4 gene expression levels in dataset from kidneys of unilaterally ureteral obstruction (UUO) mouse models (GSE261665). **(I)** Gene set enrichment analysis (GSEA) enrichment plot of CHD4 target genes (GSE261665). Data are presented as mean ± SEM. *p < 0.05, **p < 0.01, ***p < 0.001, one-way ANOVA with Bonferroni’s post-test **(C–E)**, or Student’s t test **(F-H)**.

**FIGURE 6:**
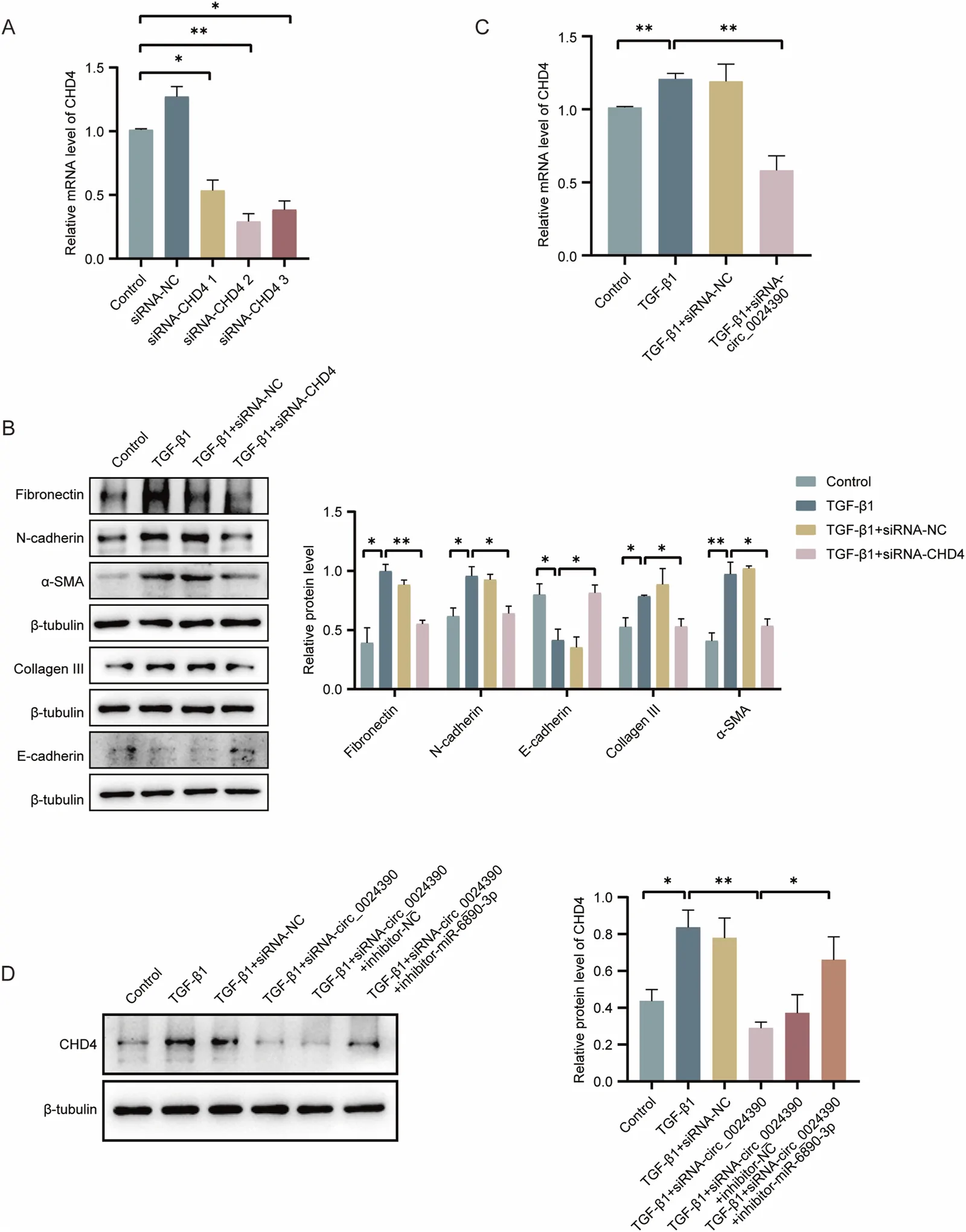
Circ_0024390 sponges miR-6890-3p to regulate CHD4 expression. **(A)** RT-qPCR analysis of CHD4 expression in HK-2 cells transfected with siRNA targeting CHD4 (n = 3). **(B)** Representative Western blot images and quantitative analysis of Fibronectin, N-cadherin, E-cadherin, Collagen III, and α-SMA protein levels (n = 3). **(C)** RT-qPCR analysis of CHD4 expression in HK-2 cells transfected with siRNA targeting circ_0024390 (n = 3). **(D)** Representative Western blot images and quantitative analysis showing that circ_0024390 increases CHD4 expression through miR-6890-3p (n = 3). Data are presented as mean ± SEM. *p < 0.05, **p < 0.01, ***p < 0.001, one-way ANOVA with Bonferroni’s post-test **(A–D)**.

## 4 Discussion

RIF is a common pathological endpoint of progressive CKD that leads to ESRD, driven by crosstalk among tubular epithelial cells, fibroblasts, endothelial cells, and infiltrating immune cells in the injured kidney (Liu, 2011). Injured tubular epithelial cells release chemokines and pro-fibrotic/pro-inflammatory mediators to recruit macrophages and T lymphocytes. Immune-cell-derived cytokines, including TGF-β1 and TNF-α, promote tubular epithelial–mesenchymal transition, myofibroblast activation, and extracellular matrix accumulation, forming an inflammation–fibrosis positive feedback loop that remodels the interstitial microenvironment and disrupts renal structure and function (Guo et al., 2023; Meng et al., 2016). In this study, circ_0024390 was markedly upregulated in TGF-β1-stimulated HK-2 cells and exacerbated RIF progression by promoting EMT in renal tubular cells. Specifically, circ_0024390 functions as a competing endogenous RNA (ceRNA) by sponging miR-6890-3p, thereby upregulating CHD4 expression. These results suggest that targeting circ_0024390 or administering miR-6890-3p mimics may represent promising therapeutic strategies for halting RIF progression in patients with CKD.

Due to their unique circular structure, circRNAs can function as ceRNAs by binding specific miRNAs, thereby attenuating the inhibitory effect of miRNAs on their target genes. This regulatory mechanism, involving specific circRNAs acting as ceRNAs, has been widely reported in various kidney diseases such as acute kidney injury (AKI), diabetic kidney disease (DKD), lupus nephritis (LN), and renal cell carcinoma (RCC) (Wang et al., 2024; Peng et al., 2021). In DKD, for example, circ_0000953 was shown to modulate podocyte autophagy by targeting miR-665-3p/Atg4b axis (Liu et al., 2024). Similarly, in AKI, circ_45478 was found to upregulate PHLPP1 expression by sponging miR-190a-5p, and silencing circ_45478 alleviated AKI progression (Liao et al., 2022). In LN, circ_0123,190 may regulate APLNR expression by acting as a ceRNA for miR-483-3p (Zhang et al., 2021). These studies highlight the crucial role of circRNAs as miRNA sponges and suggest their potential as therapeutic targets in kidney diseases. In the present study, we found that circ_0024390 was substantially elevated in TGF-β1-induced HK-2 cells. Knockdown of circ_0024390 reduced the accumulation of mesenchymal markers such as Fibronectin, Collagen III, N-cadherin, and α-SMA, while enhancing the epithelial marker E-cadherin. Furthermore, we observed that silencing circ_0024390 increased the expression of miR-6890-3p. Sequence analysis revealed that circ_0024390 contains binding sites for miR-6890-3p, indicating that miR-6890-3p is a potential target. This was further confirmed by dual-luciferase reporter assay, which demonstrated direct binding between circ_0024390 and miR-6890-3p. Consistently, overexpression of miR-6890-3p in HK-2 cells inhibited EMT, mirroring the effects of circ_0024390 knockdown. These results collectively indicate that circ_0024390 promotes EMT in HK-2 cells by acting as a ceRNA for miR-6890-3p. Of note, our data only suggest that this regulatory pathway may occur in HK-2 cells; however, we cannot rule out the possibility that it occurs in other cell types, such as fibroblasts and vascular endothelial cells.

CHD4 is an important chromatin remodeling protein and a member of the chromodomain helicase DNA-binding (CHD) protein family (Wu et al., 2023). CHD4 has been implicated in multiple pathological processes, including cell cycle dysregulation, hypoxia-related signaling pathways, excessive glycolysis, EMT, and the maintenance of tumor cell stemness (Coassolo et al., 2021; Fu et al., 2011; Zhang et al., 2022; Polo et al., 2010; Wang et al., 2020). Previous studies have demonstrated that CHD4 promotes the migration, invasion, and EMT of colorectal cancer cells (Luo et al., 2024). In a model of cholestatic liver fibrosis, CHD4 was shown to regulate β1 integrin-mediated fibrotic signaling by suppressing RIPK3 expression, thereby influencing the balance between inflammation and necrotic apoptosis (Sun et al., 2023). Moreover, CHD4 was reported to induce EMT in gastric cancer through the MYH9/GSK3β/β-catenin signaling axis (Shi et al., 2025). Our study identified CHD4 as a direct target of miR-6890-3p. Treatment of HK-2 cells with miR-6890-3p mimic significantly reduced CHD4 expression. In addition, bioinformatics analysis of publicly available datasets coupled with GSEA demonstrated a significant positive correlation between CHD4 expression and EMT related gene signatures. CHD4 knockdown inhibited the expression of EMT-associated markers, including Fibronectin, Collagen III, and N-cadherin. The indispensable role of tubular–immune cell interplay in kidney disease (Bai et al., 2024). CHD4 can regulate the differentiation of immune cells and the expression of inflammatory genes (Tomofuji et al., 2020; Hosokawa et al., 2013), suggesting that, in addition to regulating the renal tubular epithelial cells’ own EMT and ECM deposition, the circ_0024390/miR-6890-3p/CHD4 axis may also mediate crosstalk between renal tubules and immune cells, thereby contributing to the establishment and maintenance of a pro-fibrotic renal microenvironment. While our results confirm CHD4 as a direct target of miR-6890-3p, future work is needed to elucidate the precise downstream targets of CHD4 that mediate the fibrotic response, such as its interaction with other transcription factors or signaling pathways. These results suggest a potential role for the miR-6890-3p/CHD4 axis in regulating EMT.

However, our study has some limitations. First, the findings are mainly based on *in vitro* cell models. As previously reported, advanced *in vivo* models are indispensable for verifying fibrotic mechanisms and renal tissue repair activity (Xu et al., 2024). The absence of *in vivo* validation indicates that our findings provide only preliminary cellular mechanistic evidence, and the physiological and clinical significance of this axis in RIF requires further *in vivo* confirmation. Second, in this work, we attributed the pro-fibrotic function of the circ_0024390/miR-6890-3p/CHD4 axis exclusively to EMT. However, we cannot rule out the involvement of other regulated cell death mechanisms that are now recognized as key drivers of organ fibrosis. In particular, ferroptosis, a form of iron-dependent oxidative cell death, has been repeatedly shown to promote tubular injury and fibrotic progression in various kidney disease models (Lai et al., 2024). Similarly, pyroptosis, necroptosis, and apoptosis may also be functionally linked to this axis. The mechanistic scope of this study therefore warrants further expansion. Third, we did not assess circ_0024390 expression in renal biopsy samples from patients, which limits the translational relevance of our findings. Therefore, the proposed circ_0024390/miR-6890-3p/CHD4 axis warrants further investigation and validation.

In summary, our findings demonstrate that circ_0024390 promotes renal tubular EMT by targeting the miR-6890-3p/CHD4 axis, thereby exacerbating the progression of RIF (Figure 7). These results indicate that circ_0024390 may serve as a novel therapeutic target for the prevention and treatment of EMT and RIF.

**FIGURE 7:**
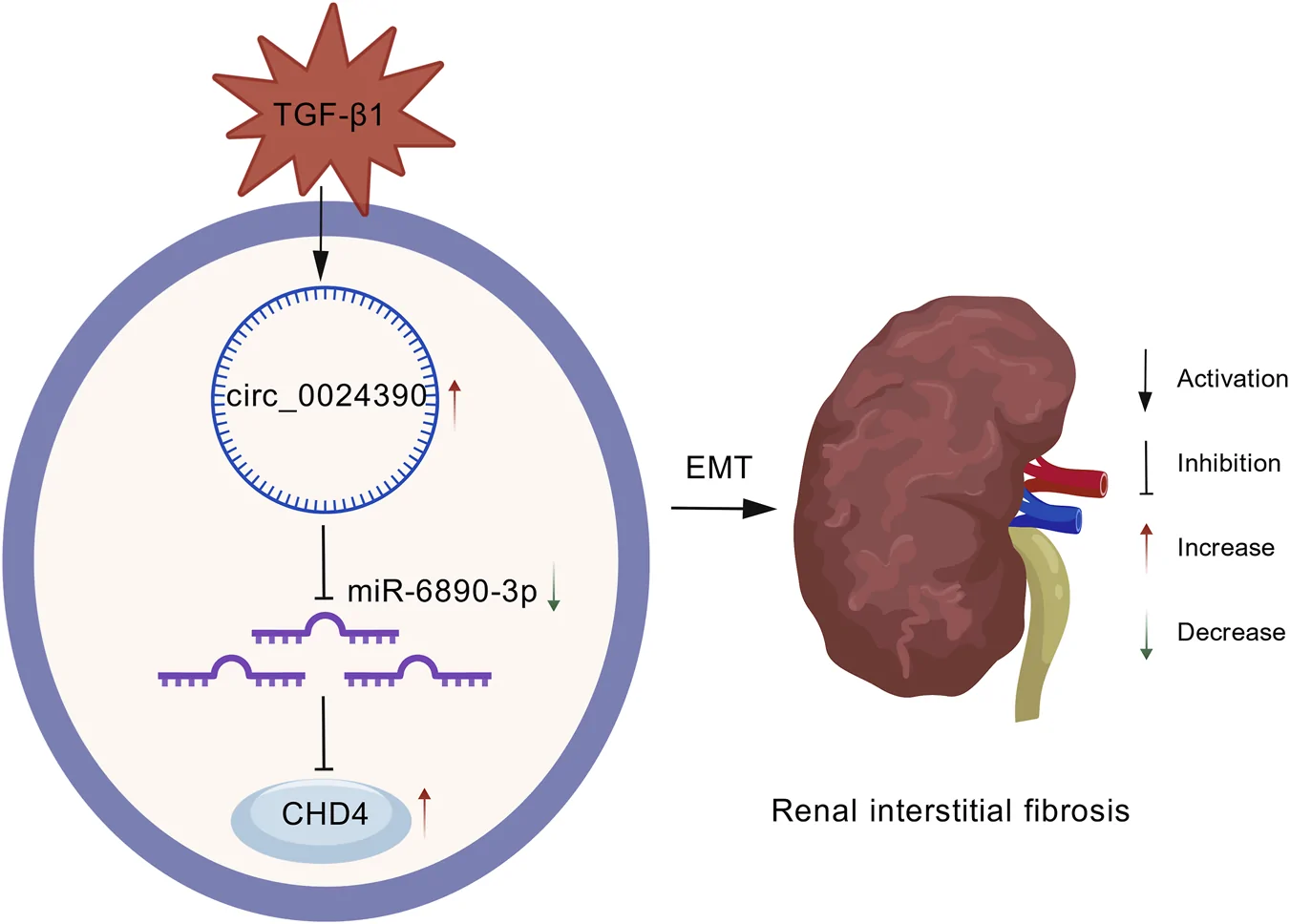
Schematic representations of the circ_0024390 regulatory network in RIF. The diagram illustrates the circ_0024390/miR-6890-3p/CHD4 regulatory axis in TGF-β1-induced EMT and the pathogenesis of RIF.

## Data Availability

The datasets presented in this study can be found in online repositories. The names of the repository/repositories and accession number(s) can be found in the article/Supplementary Material.
